# Evaluation of the Efficacy of Ozone Therapy on Liver Tissue in the Treatment of Sepsis in Rats with Cecal Perforation

**DOI:** 10.3390/medicina60091552

**Published:** 2024-09-22

**Authors:** Selin Erel, Ayşegül Küçükk, Kürşat Dikmen, Esin Tekin, Zeynep Yığman, Müşerref Şeyma Ceyhan, Seda Gökgöz, Hasan Bostancı, Mustafa Arslan, Mustafa Kavutcu

**Affiliations:** 1 Department of Anesthesiology and Reanimation, Gazi University, Ankara 06560, Turkey; selinerel@yahoo.com (S.E.); dresintekin@outlook.com (E.T.); 2 Department of Physiology, Faculty of Medicine, Kutahya Health Sciences University, Kutahya 43020, Turkey; kucukaysegul@hotmail.com; 3 Department of General Surgery, Faculty of Medicine, Gazi University, Ankara 06560, Turkey; kursatdikmen@yahoo.com (K.D.); hasanbostanci@yahoo.com (H.B.); 4 Department of Histology and Embryology, Faculty of Medicine, Gazi University, Ankara 06560, Turkey; zeynepyigman@gmail.com (Z.Y.); msceyhan@gmail.com (M.Ş.C.); 5 Neuroscience and Neurotechnology Center of Excellence NÖROM, Gazi University, Ankara 06560, Turkey; 6 Department of Medical Biochemistry, Faculty of Medicine, Gazi University, Ankara 06560, Turkey; seda.gokgoz15@gmail.com (S.G.); kavutcu@gazi.edu.tr (M.K.); 7 Life Sciences Application and Research Center, Gazi University, Ankara 06560, Turkey; 8 Laboratory Animal Breeding and Experimental Research Center (GUDAM), Gazi University, Ankara 06560, Turkey

**Keywords:** inflammation, oxidative stress, ozone, sepsis

## Abstract

*Background and Objectives:* Sepsis and its related complications are associated with high morbidity and mortality, often leading to liver damage. Ozone, a molecule with anti-inflammatory and antioxidant properties, may offer protective effects. This study aimed to evaluate the therapeutic and protective impact of ozone on liver injury in a rat model of sepsis induced by cecal ligation and perforation (CLP). *Material and Methods:* A total of 36 rats were randomly divided into five groups: control (Group C), ozone (Group O), cecal ligation and perforation (Group CLP), ozone + cecal ligation and perforation (Group O+CLP), and cecal ligation and perforation + ozone (Group CLP+O). In the ozone groups, 4 mL of ozone (20 µ/mL) was injected intraperitoneally. Biochemical and histopathological parameters were evaluated in liver tissue samples obtained at the end of 24 h. *Results:* Polymorphonuclear leukocyte and monocyte infiltration and the total injury score were significantly reduced in the ozone-treated groups compared to the CLP group (*p* < 0.001). Tumor necrosis factor and interleukin 10 levels in the rat liver tissue were significantly reduced in the O+CLP and CLP+O groups compared to the CLP group, with the O+CLP group showing a more substantial decrease than the CLP+O group (*p* < 0.001). Thiobarbituric acid reactive substances and glutathione s-transferase levels were significantly lower in the ozone-treated groups compared to the CLP group (*p* < 0.001). Catalase activity was significantly elevated in the O+CLP group compared to the CLP group (*p* < 0.001). Serum aspartate transaminase, alanine transaminase, gamma-glutamyl transferase, and total bilirubin were significantly increased in the CLP group and decreased in the ozone-treated groups (*p* < 0.001, *p* < 0.001, *p* = 0.01, *p* < 0.001 respectively). *Conclusions:* Administering ozone to rats one hour before the CLP significantly mitigated liver damage, showing a more pronounced effect compared to administering ozone one hour after CLP. The results indicate that ozone could serve a protective function in managing sepsis-induced liver damage.

## 1. Introduction

Sepsis is a critical medical condition characterized by significant organ impairment, which stems from an inadequate response to an infection [1]. It represents a significant global public health issue and is the primary driver of multiple organ dysfunction syndrome, which is linked to high in-hospital mortality rates [2,3]. Intraabdominal infections are particularly associated with elevated mortality, frequently progressing to sepsis and septic shock, and are identified as the third most frequent cause of sepsis and the second leading cause of death in intensive care settings [4,5].

The liver plays a crucial role in the immune response during critical illness due to its extensive immune-modulatory functions. In normal circumstances, the liver functions as a secondary line of defense against pathogens, following the intestines mucosal barrier [6]. Patients experiencing liver failure during sepsis frequently face long-term consequences of hepatic dysfunction, as the initial inflammatory reaction can progress to chronic liver failure [7]. In recent decades, approximately 50% of long-term mortality in sepsis patients has been attributed to complications from cholestatic liver failure [8]. Therefore, protecting the liver during sepsis could be crucial for improving survival rates.

Colonic ischemia frequently leads to colitis in the elderly. However, isolated ischemic necrosis and perforation of the cecum are uncommon and typically linked to factors such as chronic cardiac conditions, systemic sepsis, opportunistic fungal infections, hypovolemic shock, and rheumatic fever [9,10]. There are three common approaches for modeling sepsis in animal experiments: lipopolysaccharide-induced sepsis, intravenous or intraperitoneal administration of live bacteria, and the cecal ligation and perforation (CLP) model [11]. The CLP model, which involves ligation and puncture of the cecum to induce polymicrobial bacterial peritonitis, is widely regarded as the gold standard due to its reproducibility and ability to simulate sepsis characteristics that closely resemble those observed in humans. This method was chosen for our study because of its established effectiveness in mimicking human sepsis.

Ozone (O_3_) is a gas that induces antioxidant stress, increases resistance to oxidative stress, and is used as an antioxidant agent in inflammation. In medical procedures, 1–5% ozone in 95–90% oxygen is used as a gas mixture. The antibacterial, anti-inflammatory, and antioxidant properties of medical ozone treatment make it a popular therapeutic option [12].

Since sepsis is a serious illness that often results in mortality in critical care units, it is imperative that we learn more about its pathophysiology and create efficient treatment strategies. The effects of many agents, such as metformin, olmesartan, gabexate mesylate, and folic acid, on sepsis induced by CLP model have been investigated [13,14,15,16]. Different from treatments with pharmacological drugs, ozone therapy is a treatment method that strengthens the body against diseases by using antioxidant and anti-inflammatory pathways, which are the body’s own strong potentials, and not through the drug–receptor relationship [17,18].

This study investigates the therapeutic and preventive effects of ozone on the liver in rats with sepsis induced by the CLP model.

## 2. Materials and Methods

### 2.1. Experimental Animals

This study was carried out in full compliance with the regulations set forth by the National Institutes of Health. The experimental procedures followed were those authorized by the Animal Research Ethics Committee of Gazi University’s Faculty of Medicine in Ankara, Türkiye (Protocol Number: G.Ü.E.T; 23.097, approved on 26 September 2023).

In the study, 36 male Long Evans rats weighing between 250 and 350 gr were used. The rats were kept at a temperature of 20–21 °C, with 12 h of night and 12 h of daytime, and fed until 2 h before anesthesia.

### 2.2. Animal Groups and Study Design

Thirty-six rats were randomly assigned to five groups: control (Group C), ozone (Group O), cecal ligation and puncture (Group CLP), ozone administered one hour before CLP (Group O+CLP), and ozone administered one hour after CLP (Group CLP+O) (Table 1).

### 2.3. Anesthesia and Surgical Protocol

Anesthesia was induced with an intramuscular injection of 50 mg/kg of ketamine hydrochloride (Ketalar^®^, Pfizer, Istanbul, Turkey) combined with 10 mg/kg of xylazine hydrochloride (Alfazyne^®^ 2% vial, Ege Vet, Kemalpaşa/İzmir, Turkey). All surgical interventions were conducted under aseptic conditions, with the rats undergoing aseptic skin preparation and being positioned supine under a heating lamp.

Group C: Anesthesia was administered, followed by a laparotomy. The cecum was isolated without any perforation, puncture, or ligation. The abdominal cavity was then surgically closed

Group O: Following anesthesia, a laparotomy was performed. Similar to the control group, the cecum was isolated without perforation, puncture, or ligation. The abdomen was closed, and 4 mL of ozone (20 µg/mL) was administered via intraperitoneal injection

Group CLP: After anesthesia, a laparotomy was carried out, the cecum was distended with stool, ligated with 3/0 silk just below the ileocecal valve, and punctured twice on the anterior surface using an 18-gauge Intraket needle. The abdomen was subsequently surgically closed.

Group O+CLP: An intraperitoneal injection of 4 mL of ozone (20 µg/mL) was administered. One hour later, anesthesia was induced, followed by a laparotomy and CLP. The abdominal cavity was then surgically closed.

Group CLP+O: Anesthesia was induced, and CLP was performed as previously described. The abdomen was surgically closed, followed by the administration of 4 mL of ozone (20 µg/mL) via intraperitoneal injection one hour later.

### 2.4. Euthanasia and Tissue Removal

After 24 h, the rats were euthanized by intracardiac blood sampling [19]. Liver tissue specimens were excised for subsequent biochemical and histopathological analyses. Immunostaining intensity assessments for tumor necrosis factor (TNF-α) and interleukin 10 (IL-10) were conducted, and biochemical assays for thiobarbituric acid reactive substances (TBARS), catalase, and glutathione s-transferase (GST) were performed to determine the antioxidant and oxidant status.

The serum aspartate transaminase (AST), alanine transaminase (ALT), gamma-glutamyl transferase (GGT), total bilirubin, direct bilirubin, and albumin levels were analyzed from the blood samples.

### 2.5. Histological Analyses

For the histological studies, liver tissue samples of the animals were taken and fixed in 10% buffered formalin for 48 h. After fixation, the liver specimens were subjected to a routine paraffin tissue processing procedure. For this purpose, the tissues were dehydrated through a series of increasing grades of ethyl alcohol and cleared in xylene. The tissue samples were then left to be infiltrated with liquid paraffin and embedded in paraffin to obtain paraffin blocks. The paraffin blocks were sectioned with a microtome (Leica RM2245, Nussloch, Baden-Württemberg, Germany) at a thickness of 5 µm, dried at room temperature, and placed in an oven at 60 °C overnight. The tissue sections were then deparaffinized in xylene, rehydrated through a series of decreasing grades of ethyl alcohol, and prepared for hematoxylin and eosin (H&E) staining and immunostaining.

### 2.6. H&E Staining and Evaluation of Stained Sections

Liver sections were stained with H&E to evaluate the histopathological changes. For this purpose, the sections were incubated in hematoxylin and eosin solutions, each for 11 min at 25 °C. The stained liver sections were examined under a computer-assisted light microscope (Leica DM 4000B, Wetzlar, Hessen, Germany) at 200× and 400× magnifications, and images were taken using the Leica LAS V4.12 software.

To assess liver injury, the entire cross-sectional area was examined for histopathologic changes, including congestion, edema, polymorphonuclear leukocyte and monocyte infiltration, and necrosis. These changes were scored from 1 to 4, respectively. A score of 1 was given for congestion, 2 for edema, 3 for polymorphonuclear leukocyte and monocyte infiltration, and 4 for necrosis. Accordingly, liver injury was determined with a total score ranging from 0 to 10 [20].

### 2.7. Immunostaining Procedure and Evaluation of Immunostained Sections

To assess the TNF-α and IL10 expressions of the liver specimens, 5 µm-thick paraffin sections were immunolabelled with anti-TNF-α and anti-IL10 primary antibodies. Following deparaffinization and rehydration, heat-induced antigen retrieval was conducted using a citrate buffer (pH 6.0). Subsequently, the sections were incubated in a 3% hydrogen peroxide solution for 30 min at 25 °C to inhibit endogenous peroxidase activity. In order to avoid non-specific binding of antibodies, the sections were incubated with UltraV block (TA-125-UB, Thermo Scientific, Waltham, MA, USA) for 30 min at room temperature. Incubation of the liver sections with anti-TNF-α (1:100, E-AB-33121 Elabscience, Houston, TX, USA) and anti-IL10 (1:100, BS-0698R, Bioss-USA, Woburn, MA, USA) primary antibodies at +4 °C overnight was followed by the incubation with the biotinylated secondary antibody (TP-125-BN, Thermo Scientific, USA) for 2 h at 25 °C. Then, the sections were subjected to streptavidin peroxidase enzyme complex (TS-125-HR, Thermo Scientific, USA) for 30 min at 25 °C. To visualize the immunoreactivity, diaminobenzidine (DAB) chromogen-substrate complex (TA-125-HDX, Thermo Scientific, USA) was used. Immunostained sections were examined under a computer-assisted light microscope (Leica DM 4000B, Germany) at 200× magnification, and images were taken using Leica LAS V4.12 software. To assess the TNF-α and IL10 immunostaining intensity of the liver sections, micrographs of six randomly chosen non-overlapping fields at 200× magnification from each specimen were analyzed, and the immunostaining intensity of each field was calculated according to the formula H-Score = ΣP_i_ (i + 1) [21]. To perform the H-Score measurements, ImageJ software (1.53v; National Institutes of Health) was used [22].

### 2.8. Biochemical Analyzes

Liver tissues were first rinsed with a chilled 0.154 M sodium chloride solution to remove contaminants, and then homogenized for about 3 min at 1000 U using a Diax 900 homogenizer (Heidolph Instruments GmbH & Co. KG, Schwabach, Germany). After centrifugation at 10,000× *g* for about one hour, the supernatant clear layer was collected.

To measure the TBARS levels, the thiobarbituric acid reactive substances assay was conducted following the method of Van Ye et al. [23]. The reaction with thiobarbituric acid at 80–90 °C was employed to determine the TBARS levels, as malondialdehyde or similar substances react with thiobarbituric acid, producing a pink pigment with a maximum absorption at 532 nm. To facilitate protein precipitation, the sample at 25 °C was mixed with chilled 20% (*w*/*v*) trichloroacetic acid, and the precipitate was then centrifuged for 10 min at 3000 RPM at 25 °C to form a pellet. An aliquot of the supernatant was subsequently placed into an equal volume of 0.6% (*w*/*v*) thiobarbituric acid and incubated in a boiling water bath for 30 min. After cooling, the absorbance of both the sample and the blank was measured at 532 nm, with the results reported in nmol/mg of protein. These values were derived from a calibration curve using 1,1,3,3-tetramethoxypropane as the malondialdehyde standard.

The catalase activity was assessed by measuring the decrease in absorbance due to hydrogen peroxide consumption, as described by Aebi H [24].

The GST enzyme activity was measured based on the method of Habig et al., tracking absorbance increases at 340 nm due to DNPG reduction [25]. The results were expressed in IU/mg protein using the molar extinction coefficient of the DNPG complex.

### 2.9. Statistical Analysis

The data obtained were analyzed using Statistical Package for the Social Sciences (SPSS, version 22.0, IBM Corp., Armonk, NY, USA). The normality was assessed visually (histograms, probability graphs) and analytically (Kolmogorov–Smirnov/Shapiro–Wilk tests). Statistical analysis involved the Kruskal–Wallis test, Mann–Whitney U test with Bonferroni correction, or one-way ANOVA with Tukey’s test. The results were reported as the mean ± standard deviation (SD) and median (interquartile range (IQR)). The total type I error level was used as 5% for statistical significance; *p* < 0.05 is statistically significant.

## 3. Results

### 3.1. Results of the Evaluation of H&E-Stained Sections

**Figure 1:**
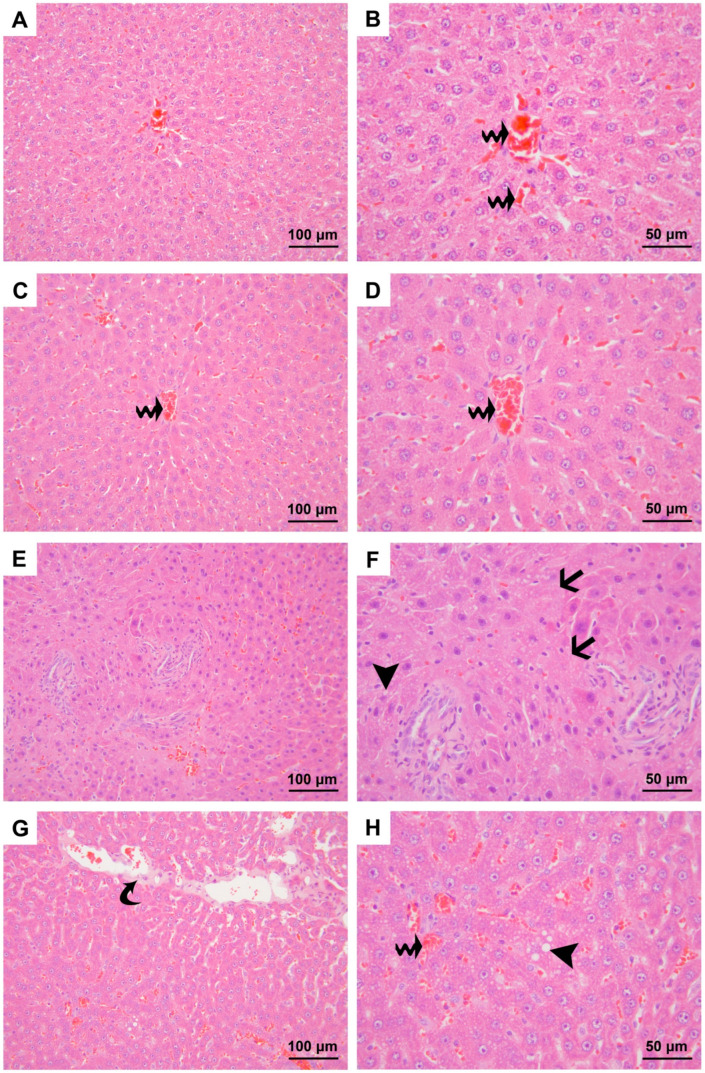
Micrographs representing the H&E-stained sections of specimens from the control (**A**,**B**), ozone (**C**,**D**), and CLP (**E**–**H**) groups. Black arrow, necrosis; black arrowhead, varying levels of vacuolization in hepatocytes; wavy black arrow, congestion; curved black arrow, interstitial edema. Magnifications are 200× for micrographs (**A**,**C**,**E**,**G**) and 400× for (**B**,**D**,**F**,**H**). Stain: H&E.

**Figure 2:**
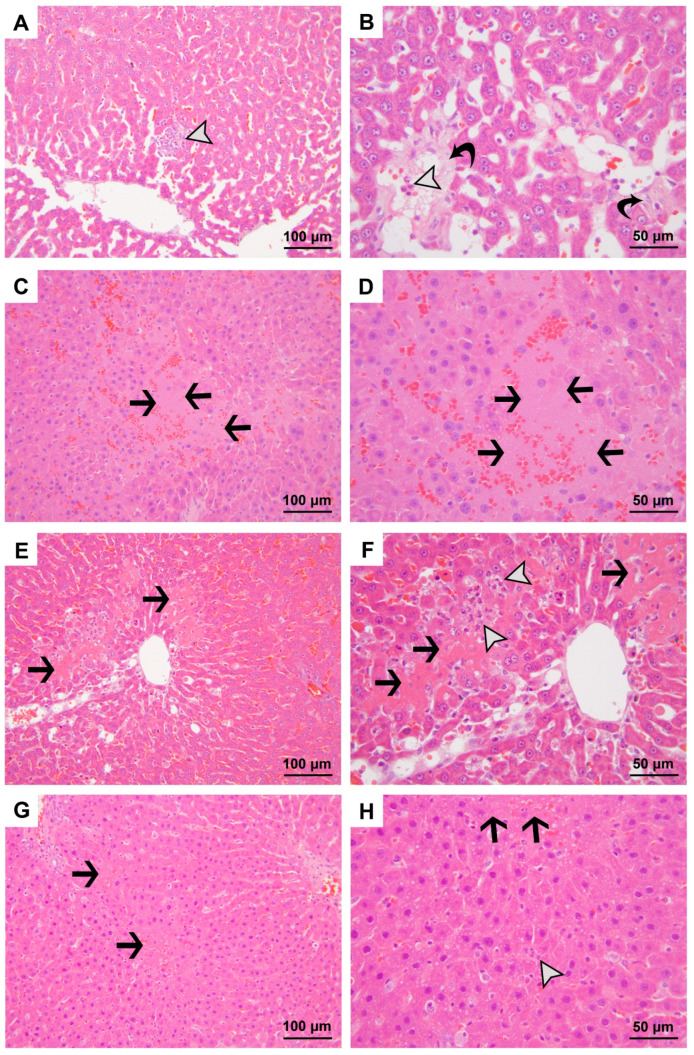
Micrographs representing the H&E-stained sections of specimens from the CLP (**A**–**D**), O+CLP (**E**,**F**), and CLP+O (**G**,**H**) groups. Black arrow, necrosis; white arrowhead, polymorphonuclear leukocyte and monocyte infiltration; curved black arrow, interstitial edema. Magnifications are 200× for micrographs (**A**,**C**,**E**,**G**) and 400× for micrographs (**B**,**D**,**F**,**H**). Stain: H&E.

Histological examination of the H&E-stained liver samples revealed significant differences among the experimental groups in terms of edema, polymorphonuclear leukocyte and monocyte infiltration, necrosis, and total liver injury score (*p* < 0.001). There were no significant differences observed in the degree of congestion among the groups (Table 2, Figure 1 and Figure 2).

Significant edema was observed in the CLP, O+CLP and CLP+O groups compared to Group C. Edema was more pronounced in the CLP and O+CLP groups compared to Group O, while it was significantly lower in Group CLP+O compared to Group CLP (Table 2, Figure 1 and Figure 2).

Polymorphonuclear leukocyte and monocyte infiltration significantly increased in Group CLP and Group CLP+O compared to Group C and Group O. Conversely, infiltration was significantly lower in the O+CLP and CLP+O groups compared to Group CLP (Table 2, Figure 1 and Figure 2).

Necrosis and the total liver damage score were significantly higher in the CLP, O+CLP, and CLP+O groups compared to Groups C and O (Table 2, Figure 1 and Figure 2). However, the total liver damage score was lower in Group O+CLP and Group CLP+O compared to Group CLP.

### 3.2. Results of the Evaluation of Immunostained Sections

Significant differences were also observed between the experimental groups regarding TNF-α and IL10 immunostaining intensities of rat liver tissue samples (*p* < 0.001) (Table 3).

**Figure 3:**
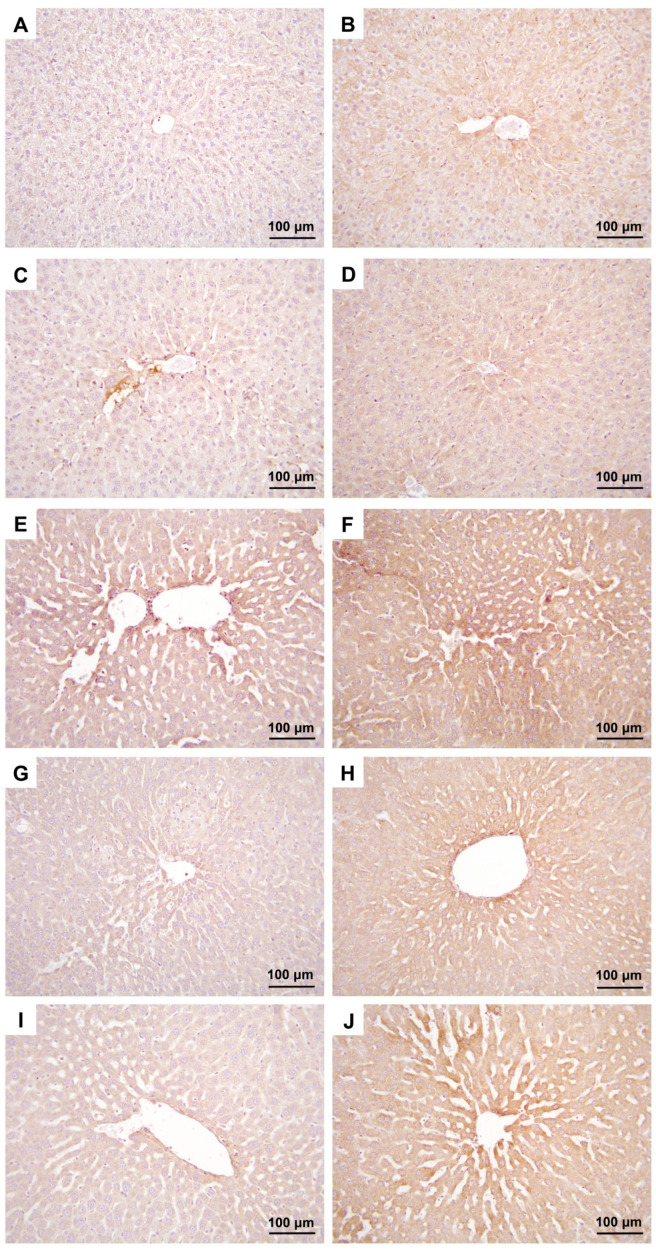
Micrographs (**A**,**C**,**E**,**G**,**I**) represent the IL10 immunostaining intensity of specimens from the control (**A**), ozone (**C**), and CLP (**E**), O+CLP (**G**), and CLP+O (**I**) groups. Micrographs (**B**,**D**,**F**,**H**,**J**) represent the TNF-α immunostaining intensity of specimens from the control (**B**), ozone (**D**), and CLP (**F**), O+CLP (**H**), and CLP+O (**J**) groups. Magnification is 200× for all micrographs.

The IL10 immunostaining intensity in the CLP group was significantly higher than those of the C and O groups, while the IL10 immunostaining intensity in the O+CLP and CLP+O groups was significantly lower compared to the CLP group (Table 3, Figure 3).

When the groups were compared in terms of the TNF-α immunostaining intensity, it was found to be significantly higher in the CLP group than those in the C and O groups. Meanwhile, the TNF-α immunostaining intensity was significantly higher in the O+CLP and CLP+O groups in comparison to the C and O groups, TNF-α immunoreactivity was significantly lower in the O+CLP and CLP+O groups compared to the CLP group (Table 3, Figure 3).

### 3.3. Results of the Biochemical Analyses

When comparing the oxidative status parameters in the liver tissues among the groups, significant differences were found. The TBARS levels were markedly higher in the CLP group compared to Group C (*p* < 0.0001). Additionally, the TBARS levels were significantly lower in the CLP+O and O+CLP groups compared to the CLP group (Table 4).

The catalase enzyme activity was significantly lower in the CLP group compared to Group C (*p* < 0.0001), but notably higher in the CLP+O and O+CLP groups compared to the CLP group. The GST enzyme activity was significantly elevated in the CLP group compared to Groups C and O (*p* < 0.0001). However, the GST activity was significantly reduced in the CLP+O and O+CLP groups compared to the CLP group (Table 4).

The serum biochemical parameters also revealed significant differences among the groups. The serum ALT, AST, GGT, total bilirubin, and direct bilirubin levels were significantly higher in the CLP group (*p* < 0.001, *p* < 0.001, *p* = 0.014, *p* < 0.001, and *p* = 0.002, respectively). In the ozone-treated groups, the serum ALT, AST, GGT, total bilirubin, and direct bilirubin levels were significantly lower in the CLP+O group, with more pronounced reductions in the O+CLP group. The albumin levels were decreased in the CLP group compared to Groups O and C. However, the albumin levels were higher in the O+CLP group compared to the CLP group (Table 5).

## 4. Discussion

Our findings indicate that ozone reduced the TBARS and GST levels, which are indicators of oxidative stress, and enhanced the activity of catalase, an antioxidant enzyme. Additionally, ozone administration led to decreased the levels of ALT, AST, GGT, and both the total and direct bilirubin, while increasing the albumin levels, reflecting improved liver function. Histopathological analysis revealed that the ozone treatment lessened edema, necrosis, and the infiltration of polymorphonuclear leukocytes and monocytes in the liver tissue, thereby mitigating liver damage associated with sepsis. Beyond its beneficial effects on liver tissue, ozone also decreased the levels of interleukin-10, an anti-inflammatory cytokine, and TNF-alpha, a pro-inflammatory cytokine involved in the acute phase response.

High levels of reactive oxygen and nitrogen species are produced during sepsis, and this can result in multiple organ damage [16]. Sepsis-related liver damage can be lethal; therefore, comprehending the liver’s function is essential to understanding the elaborate pathophysiology of sepsis. The liver modulates immune function by secreting cytokines that modify the immune response and boost adaptive immunity. Moreover, liver damage significantly affects overall metabolism, as well as both natural and acquired immune functions, due to the liver’s role to release pro- and anti-inflammatory substances and to sequester immune-regulating endogenous and exogenous substances [26,27].

The movement of electrons inside the mitochondria during hepatocyte cell injury gradually raises the possibility of producing reactive oxygen species, such superoxide, hydrogen peroxide, and hydroxyl radicals [28]. As demonstrated in our study, this pathological chain reaction subjects the liver to significant oxidative stress, potentially leading to the death of hepatocytes through necrosis [29].

In animal models of liver damage, exposure to ozone has been shown to have positive benefits by decreasing tissue oxidative stress and inflammatory marker expression, which in turn helps to mitigate pathological processes, including necrosis and degeneration. In addition, exposure to ozone suppresses the NF-κB inflammatory pathway and modifies the Nrf2/ARE antioxidant pathway. This modulation efficiently controls oxidative stress and the inflammatory process by upregulating antioxidant enzyme production and downregulating pro-inflammatory cytokines, including TNF-alpha and IL-1β [30].

TNF-α is an inflammatory cytokine produced by macrophages and monocytes during acute inflammation, playing a crucial role in various cellular signaling pathways that lead to necrosis or apoptosis [30]. Ozone exposure has been shown to effectively reduce cell degeneration and necrosis [30]. It inhibits apoptotic processes by downregulating the expression of caspase, TNF-α, Bcl-2-associated protein X (Bax), and p53 genes. This effect is directly linked to the reduction in the number of inflammatory cells, such as neutrophils and Kupffer cells, following ozone exposure [30]. Zamora et al. demonstrated that ozone preconditioning, administered five days prior, significantly reduced TNF-α levels in a mice model of endotoxic shock. Similarly, in our study, TNF-α levels—an indicator of systemic inflammatory response—were elevated due to CLP but decreased following ozone treatment [31].

IL-10 is an anti-inflammatory cytokine that promotes innate immune responses from tissue epithelia, helping to limit damage caused by both viral and bacterial infections. Additionally, IL-10 plays a crucial role in facilitating tissue healing after infection or inflammation [30]. Although some studies in the literature have demonstrated that ozone influences anti-inflammatory cytokines, the findings are mixed, with some studies reporting an increase and others a decrease in IL-10 levels following ozone treatment [30]. In our study, the IL-10 levels decreased in the ozone-treated rats, suggesting a potential modulatory effect. However, we believe that further research is necessary to fully understand this relationship.

Ozone exposure can affect cellular antioxidant levels in both favorable and negative ways. It was discovered that ozone preconditioning increased cellular antioxidants in cases where hepatic injury decreased antioxidant marker levels. Ozone preconditioning has been shown to normalize the levels of catalase, glutathione peroxidase, and superoxide dismutase in certain situations when hepatic injury had caused these indicators to increase. This suggests that ozone aids in maintaining redox equilibrium and reducing tissue damage. On the other hand, ozone preconditioning reduced the high antioxidant marker levels caused by hepatic damage and brought them closer to the control values, which helped to restore redox equilibrium [30]. In an early study by Takeda et al., an elevated level of TBARS was observed in septic patients, suggesting increased lipid peroxidation [32]. In our study, both protective and therapeutic effects of ozone treatment on sepsis were demonstrated, characterized by decreased TBARS and GST levels and increased catalase levels.

In our study, ozone exposure lowered the ALT, AST, and bilirubin levels, while increasing albumin levels in sepsis-induced liver damage. Since ALT and AST are generated by hepatocytes and released only when these cells are damaged, our findings underscore the cytoprotective effect of ozone on hepatocytes, leading to a reduction in the release of ALT and AST [26]. Additionally, liver-specific bile acids serve as inducers of apoptosis and necrosis, depending on their hydrophobicity and current concentration [30]. These results demonstrate the therapeutic and protective effects of ozone.

In the existing literature, the ozone’s effects on various organs, including the lungs, colon, nerve cells, and others have been extensively studied [33,34,35,36]. While studies have demonstrated ozone’s impact on sepsis and the liver, its specific influence on the liver in septic rats has not been previously explored. Research investigating ozone’s effects on lung tissue damage in septic rats has shown significant reductions in oxidative stress and the levels of proinflammatory cytokines, alongside decreased myeloperoxidase activity and increased activity of antioxidant enzymes. Moreover, ozone administration suppressed IL-1β levels and improved histological outcomes [37]. Experimental research has also documented ozone’s beneficial effects by enhancing SOD activity and decreasing glutathione levels in models of acetaminophen-induced liver injury and liver ischemia–reperfusion injury [38,39]. Çakır et al. investigated the effects of ozone on colon anastomosis in a peritonitis model in rats and showed that intraperitoneal ozone administration decreased the TNF-α and IL-1β levels and contributed to tissue healing by increasing proliferation and vascularization [40].

Our study has limitations. Firstly, the maximum number of animals per group was limited by the ethics committee’s guidelines. Therefore, a formal sample size calculation was not performed prior to the study. However, based on previous studies utilizing similar models, a sample size of 36 animals was deemed sufficient to observe statistically significant differences in the measured outcomes. Secondly, the effects of ozone could have been more thoroughly explored by investigating different doses at various time points during the progression of sepsis, which might have allowed us to identify the optimal dose and timing of ozone administration. Future studies could address this by examining varying ozone doses at different intervals before and after the induction of sepsis using the CLP model.

## 5. Conclusions

Our study has shown, through histopathological and biochemical analyses, that ozone administration one hour prior to cecal ligation and puncture in rats effectively mitigated liver damage, exhibiting more pronounced effects compared to ozone administration one-hour post-CLP.

## Figures and Tables

**Table 1:** Animal groups and study design.

Group C	Sham (Laparotomy only)	*n* = 6
Group O	Sham+O (Laparotomy and ozone administration)	*n* = 6
Group CLP	Cecal ligation and perforation	*n* = 8
Group CLP+O	Cecal ligation and perforation + ozone (1 h after the sepsis model)	*n* = 8
Group O+CLP	Ozone + cecal ligation and perforation (1 h before the sepsis model)	*n* = 8

**Table 2:** Results of histopathological evaluations of hematoxylin and eosin-stained liver sections (median ± IQR).

	Group C (*n* = 6)	Group O (*n* = 6)	Group CLP (*n* = 8)	Group O+CLP (*n* = 8)	Group CLP+O (*n* = 8)	*p* **
Congestion	0.50 (0–1)	0.50 (0–1)	1.00 (1–1)	0.50 (0–1)	1 (0.25–1)	0.315
Edema	0.00 (0–0)	0.00 (0–2)	2.00 (2–2) *^,&^	2.00 (1.50–2) *^,&^	1.00 (0–2) *^,+^	<0.001
Polymorphonuclear leukocyte and monocyte infiltration	0.00 (0–0)	0.00 (0–0.75)	3.00 (3–3) *^,&^	1.00 (0–3)+	2.00 (0–3) *^,&,+^	<0.001
Necrosis	0.00 (0–0)	0.00 (0–0)	4.00 (4–4) *^,&^	4.00 (4–4) *^,&^	4.00 (4–4) *^,&^	<0.001
Total injury score	0.50 (0–1)	2.00 (0–3)	10.00 (10–10) *^,&^	6.50 (6–7.75) *^,&,+^	7.00 (5.25–9.50) *^,&,+^	<0.001

*p* **: Significance level *p* < 0.05 by Kruskal–Wallis test; * *p* < 0.05 compared with Group C; ^&^ *p* < 0.05 compared with Group O; ^+^ *p* < 0.05 compared with Group CLP.

**Table 3:** Results of TNF-α and IL10 immunostaining intensity assessments of liver sections (mean ± SD).

	Group C (*n* = 6)	Group O (*n* = 6)	Group CLP (*n* = 8)	Group O+CLP (*n* = 8)	Group CLP+O (*n* = 8)	*p* **
IL10	122.89 ± 5.58	119.94 ± 10.08	160.05 ± 12.48 *^,&^	134.71 ± 10.65 ^+^	135.68 ± 13.09 ^+^	<0.001
TNF-α	163.37 ± 9.37	163.01 ± 16.40	193.65 ± 9.84 *^,&^	178.42 ± 8.36 *^,&,+^	181.14 ± 7.32 *^,&,+^	<0.001

Interleukin 10 (IL10), tumor necrosis factor (TNF-α). *p* **: Significance level *p* < 0.05 by ANOVA test; * *p* < 0.05 compared with Group C; ^&^ *p* < 0.05 compared with Group O; ^+^ *p* < 0.05 compared with Group CLP.

**Table 4:** Oxidant status parameters of rat liver tissue (mean ± SD).

	Group C (*n* = 6)	Group O (*n* = 6)	Group CLP (*n* = 8)	Group O+CLP (*n* = 8)	Group CLP+O (*n* = 8)	*p* **
TBARS (nmol/mg protein)	0.08 ± 0.02	0.10 ± 0.02	0.16 ± 0.02 *^,&^	0.13 ± 0.02 *^,&,+^	0.14 ± 0.03 *^,&,+^	<0.0001
Catalase (IU/mg protein)	154.92 ± 27.42	130.35 ± 6.60	82.67 ± 23.84 *^,&^	128.62 ± 49.08 ^&,+^	102.07 ± 8.54 *^,&^	<0.0001
GST (IU/mg protein)	0.22 ± 0.06	0.20 ± 0.02	0.33 ± 0.05 *^,&^	0.23 ± 0.06 ^+^	0.27 ± 0.05 *^,&,+^	<0.0001

Thiobarbituric acid reactive substances (TBARS), glutathione s-transferase (GST). *p* **: Significance level of *p* < 0.05 by ANOVA test; * *p* < 0.05 compared with Group C; ^&^ *p* < 0.05 compared with Group O; ^+^ *p* < 0.05 compared with Group CLP.

**Table 5:** Serum AST, ALT, GGT, total bilirubin, direct bilirubin, and albumin parameters (mean ± SD).

	Group C (*n* = 6)	Group O (*n* = 6)	Group CLP (*n* = 8)	Group O+CLP (*n* = 8)	Group CLP+O (*n* = 8)	*p* **
ALT (U/L)	73.67 ± 14.58	61.00 ± 15.94	400.14 ± 182.54 *	185.17 ± 60.06 *^,&,+^	228.00 ± 46.51 *^,&,+^	<0.001
AST (U/L)	143.83 ± 17.36	173.17 ± 30.47	2065.71 ± 379.48 *^,&^	1092.83 ± 97.03 *^,&,+^	1110.63 ± 316.93 *^,&,+^	<0.001
GGT (U/L)	7.83 ± 1.47	8.33 ± 2.66	14.86 ± 4.06 *^,&^	10.83 ± 4.49 ^+^	10.62 ± 4.37 ^+^	0.014
Total bilirubin (mg/dL)	0.10 ± 0.00	0.10 ± 0.00	0.36 ± 0.14 *^,&^	0.14 ± 0.05 ^+^	0.18 ± 0.09 ^+^	<0.001
Direct bilirubin (mg/dL)	0.10 ± 0.00	0.10 ± 0.00	0.24 ± 0.11 *^,&^	0.11 ± 0.02 ^+^	0.14 ± 0.07 ^+^	0.002
Albumin (mg/dL)	4.17 ± 0.58	3.70 ± 0.46	2.90 ± 0.47 *^,&^	3.45 ± 0.29 *^,+^	3.19 ± 0.42 *	<0.001

Alanine transaminase (ALT), aspartate transaminase (AST), gamma-glutamyl transferase (GGT). *p* **: Significance level of *p* < 0.05 by ANOVA test; * *p* < 0.05 compared with Group C; ^&^ *p* < 0.05 compared with Group O. ^+^ *p* < 0.05 compared with Group CLP.

## Data Availability

The datasets used and/or analyzed during the current study are available from the corresponding author upon reasonable request.
